# Googling Social Interactions: Web Search Engine Based Social Network Construction

**DOI:** 10.1371/journal.pone.0011233

**Published:** 2010-07-21

**Authors:** Sang Hoon Lee, Pan-Jun Kim, Yong-Yeol Ahn, Hawoong Jeong

**Affiliations:** 1 Department of Physics, Korea Advanced Institute of Science and Technology, Daejeon, Korea; 2 Institute for Genomic Biology, University of Illinois at Urbana-Champaign, Urbana, Illinois, United States of America; 3 Center for Complex Network Research, Northeastern University, Boston, Massachusetts, United States of America; 4 Center for Cancer Systems Biology and Department of Cancer Biology, Dana-Farber Cancer Institute, Boston, Massachustees, United States of America; 5 Institute for the BioCentury, Korea Advanced Institute of Science and Technology, Daejeon, Korea; German Cancer Research Center, Germany

## Abstract

Social network analysis has long been an untiring topic of sociology. However, until the era of information technology, the availability of data, mainly collected by the traditional method of personal survey, was highly limited and prevented large-scale analysis. Recently, the exploding amount of automatically generated data has completely changed the pattern of research. For instance, the enormous amount of data from so-called high-throughput biological experiments has introduced a systematic or network viewpoint to traditional biology. Then, is “high-throughput” sociological data generation possible? Google, which has become one of the most influential symbols of the new Internet paradigm within the last ten years, might provide torrents of data sources for such study in this (now and forthcoming) digital era. We investigate social networks between people by extracting information on the Web and introduce new tools of analysis of such networks in the context of statistical physics of complex systems or socio-physics. As a concrete and illustrative example, the members of the 109th United States Senate are analyzed and it is demonstrated that the methods of construction and analysis are applicable to various other weighted networks.

## Introduction

Social relationships among people [1], [2] are composed of various weight of ties, as much as metabolic pathways [3] or airline traffic networks [4], [5]. However, introducing proper weight for the relationships in social networks is not an easy task since it is hard to objectively quantify the relatedness among people. As people's activities on the Web and communications via social networking service become more popular, information about the social relationships among people (especially for famous figures, through news and blog sites) becomes available and can be used as a source of high-throughput data. Here, we suggest that the ability of search engines can be used for this task. Search engines count/estimate the number of webpages including all the words in a search query, and this feature can be used to measure the relatedness between pairs of people in social networks in which we are interested. The more webpages that are found, the more popular or relevant the combination of the search query is. Therefore, *cooccurrence* of two people in many personal webpages, news articles, blog articles, Wikipedia, *etc.* on the Web implies that they are more closely related than two random counterparts.

There are several advantages of using search engines to construct social relatedness networks. First, with a list of names, one can systematically count the number of webpages containing two names simultaneously, extracted by search engines to assign the weights of all the possible pairs. This procedure enormously reduces the necessary efforts to extract social networks, compared with the traditional methods based on surveys. In addition, such automation makes analysis of enormous amount of data related to social networks possible and helps us to avoid subjective bias, such as the “self-report” format of personal surveys [6]. Furthermore, if one extracts social networks from a group of people on a regular basis over a certain period, the temporal change or stability of the relationship between group members in the period can be monitored. Although it is possible that some error or artifacts, such as several people with the same name [7], are caused by this systematic approach, this can also be managed by adding extra information (such as putting additional queries like the subjects' occupations into the search engine, in such cases). Furthermore, the *cost* of investigation with the search engine is much smaller. This example highlights the effectiveness, objectiveness, and accuracy of the usage of Web search engines.

## Materials and Methods

### Datasets and Google Correlation

Based on the pairwise correlations extracted from Google, we constructed and analyzed the weighted social networks among the Senators in the 109th United States Congress (US Senate), as well as some other social groups from academics and sports. Our datasets are three representative communities with very different characteristics, i.e., politicians, physicists, and professional baseball players. The US Senate in the 109th Congress (http://www.senate.gov) consists of 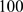 Senators, two for each state. Among the physicists who submitted abstracts to American Physical Society (APS) March Meeting 2006(Bulletin of the American Physical Society Vol. 51, No. 1. American Physical Society.), we selected the subset of 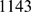 authors who submitted more than two abstracts for computational tractability. Finally, the list of Major League Baseball (MLB) players is the 40-man roster (March 28, 2006) with 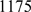 players (http://mlb.com). To avoid the ambiguous situation where there is more than one person with the same name, the following distinguishing words or phrases were added to all the search queries for each group: the words are “senator” for US Senators, “physicist” for APS authors, and “baseball” for MLB players. First, we recorded the number of pages searched using Google for each member's name, which were assigned as the Google hits [8] showing the fame of each individual member.

The *Google correlation* between two members of a group is defined as the number of pages searched using Google when the pair of members' names (and the additional word) is entered as the search query. We have removed the number of Web pages from 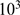 to 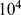 and divided the number greater than 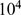 by 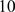, based on our judgment about the *page counting problem* in Google. See Google Inconsistencies (2003) http://www.searchengineshowdown.com/features/google/inconsistent.shtml. (last accessed on 6/2/2010) We observed that there exists an obvious *gap* between the number of 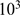 and 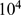 in the distributions of Google hits and correlations, and found that if we process data by the removing and dividing mentioned, the distributions become smooth without any gap. This process, however, does not cause any relevant changes in the main results of our work. In this case, Google shows the number of searched pages including *all the words in the search query*. Simply, this Google correlation value is assigned as the link's weight for the pair of nodes. If no searched page is found for a pair, the pair is not considered to be connected. Note that the idea of using co-occurrence to quantify the correlation was presented before in systems biology [9] or linguistics [10], [11], but our work comprehensively approaches such a general concept and focuses on the digital records to extract information. The constructed weighted networks are usually densely connected: the link density, defined as the ratio of existing links to all the possible links among nodes (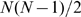, where 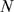 is the number of nodes), is 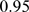 for the US Senate, 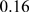 for APS authors, and 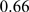 for MLB players.

**Figure 1:**
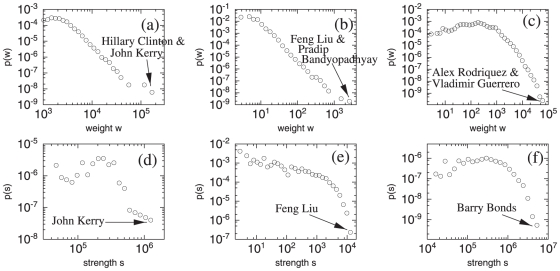
The weight and strength distributions. Google correlation value (weight) distributions 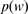 for (a) US Senate, (b) APS authors, and (c) MLB players and the strength distributions 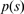 for (d) US Senate, (e) APS authors, and (f) MLB players are shown. Pairs with the largest Google correlation values (a)–(c) and the nodes with largest strengths (d)–(f) for each plot are indicated.

Due to the high link density, elaborating on the weights of links or the strength (the sum of the weights around a specific node) of nodes to extract useful information is more important. Figure 1 shows the weight and strength distributions for the weighted networks constructed by assigning the Google correlation values as link weights. Previous studies on other weighted networks show heavy tailed weight and strength distributions [4], [5] and our networks also reveal such broad distributions spanning several orders of magnitude, although the details are different for each network.

### The Rényi Disparity

**Figure 2:**
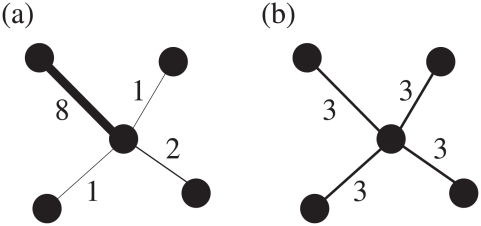
Two nodes in weighted networks with the same values of degree and strength. The degree of the central node in both (a) and (b) is 4 and the strength is 12, but the distributions of weights around the nodes are quite different.

The degree and strength are basic quantities that estimate the importance of nodes in a weighted network [4], [5]. However, the weights on the links of two nodes with the same degree and strength are not necessarily identically distributed. In other words, just the number of links a node has (degree) and the sum of weights on the links the node has (strength) are not sufficient to fully conceive the node's character. For example, two central nodes in Fig. 2 have the exactly same values of degree and strength, but the weight distributions around the nodes are totally different. Quantifying such different forms of weight distributions is important because it can distinguish whether a node's relationship with its neighboring nodes is dominated only by a small portion of neighbors or if almost all the neighbors contribute similarly to the node's relationship. As an initial step to further investigation we are interested in the *dispersion* or *heterogeneity* of weights a node bears. Although this concept of disparity is not a new one [3], [12], [13], we suggest a more general framework of such quantities based on information theory.

Suppose a node 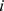 has 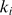 links whose weights are given by the set 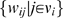, where 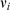 is the set of the node 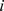
's neighboring nodes. The strength of the node is defined as 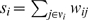. Now, let us denote 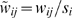 for each weight 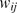 as the normalized weight. In the continuum limit of neighbor indices 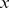 sorted by descending weights (without loss of generality) around the node 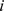 whose set of weights is 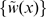, (the normalization condition becomes simply 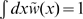 in this case) if all the neighbor indices are re-scaled as 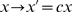 (meaning the entire network gets larger by the factor of 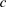, and the normalized weights become 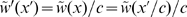 due to the normalization condition), the quantity 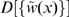 characterizing the dispersion of weights should be scaled as 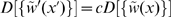. This scaling behavior is the same as the degree measure and, in fact, if all the weights are identical, the quantity is set to precisely become the degree. We have found a class of solutions satisfying such scaling conditions, which is the weighted sum
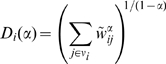
(1)to node 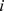, where the constant 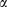 is a tunable parameter, and we denote this measure as the *Rényi disparity*. If all the weights are equal, 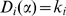, which is just the degree of node 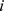, regardless of the value 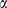. As the weight distribution deviates from the uniform distribution, 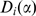 also deviates from the degree, the details of which depend on the parameter 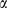, of course. We will use this weighted sum 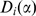 as the measure of the heterogeneity in the weight distribution for each node. Note that the logarithm of Eq. (1), 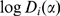, coincides with the Rényi entropy [14] in information theory, from which the name “Rényi disparity” originates. We have yet to decide the parameter 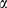 for 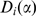. In previous works [3], [12], [13], the quantity called disparity 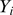 was defined for each node 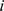. Its scaling behavior is that 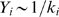 if the weights are uniformly distributed and 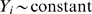 if the weight distribution is severely heterogeneous. It is easy to see that the disparity 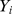 in Refs. [3], [12], [13] is the reciprocal of a special case of our Rényi disparity, with the parameter 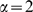, i.e.,
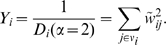
(2)The logarithm of this 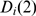 is also a special case of Rényi entropy, called the extension entropy [14], [15] and 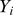 is related to the simple variance 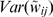 by 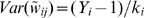.

If we consider the limiting case of 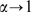, we denote it as the Shannon disparity 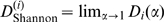 of the node 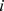. In this limit, one can easily verify that
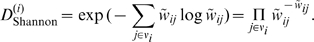
(3)One can immediately notice that the Shannon disparity is the exponential of an even more familiar and widely accepted entropy in information theory, which is the Shannon entropy [14]. The scaling property of 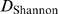 is similar to 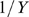 in Eq. (2) and, in fact, for our three weighted networks the two quantities 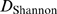 and 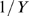 are highly correlated: the Pearson correlation coefficients are 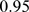 for US Senate, 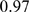 for APS authors, and 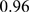 for MLB players.

Even though 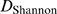 and 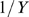 are highly correlated in our example networks, Shannon disparity works better for inhomogeneous weight distribution than the Rényi disparity with 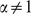. Suppose the weight around a node follows the power-law relation 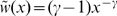 for 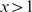, where 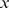 is the continuous version of the neighbor indices sorted by descending weights and the constant 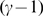 is set to the normalization condition 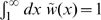.

**Figure 3:**
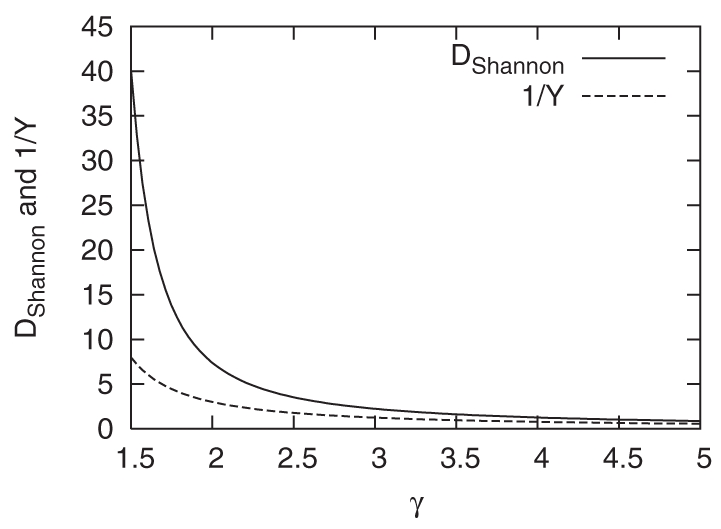
The functional form of 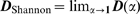 and 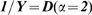 in case of the power-law weight-index relation 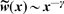, from Eqs. (4) and (5).

In this continuum limit, we can explicitly calculate the dependence of 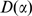 on the power-law exponent 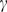 by direct integration, which is 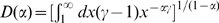. The integration is straightforward and the result is
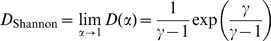
(4)

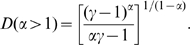
(5)As shown in Fig. 3, the Shannon disparity 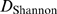 is the only Rényi disparity showing the non-polynomial scaling and more sensitive to the exponent 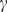 than 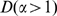, especially when 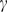 becomes smaller and 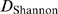 diverges much faster as 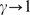 (the most homogeneous weight distribution).

**Figure 4:**
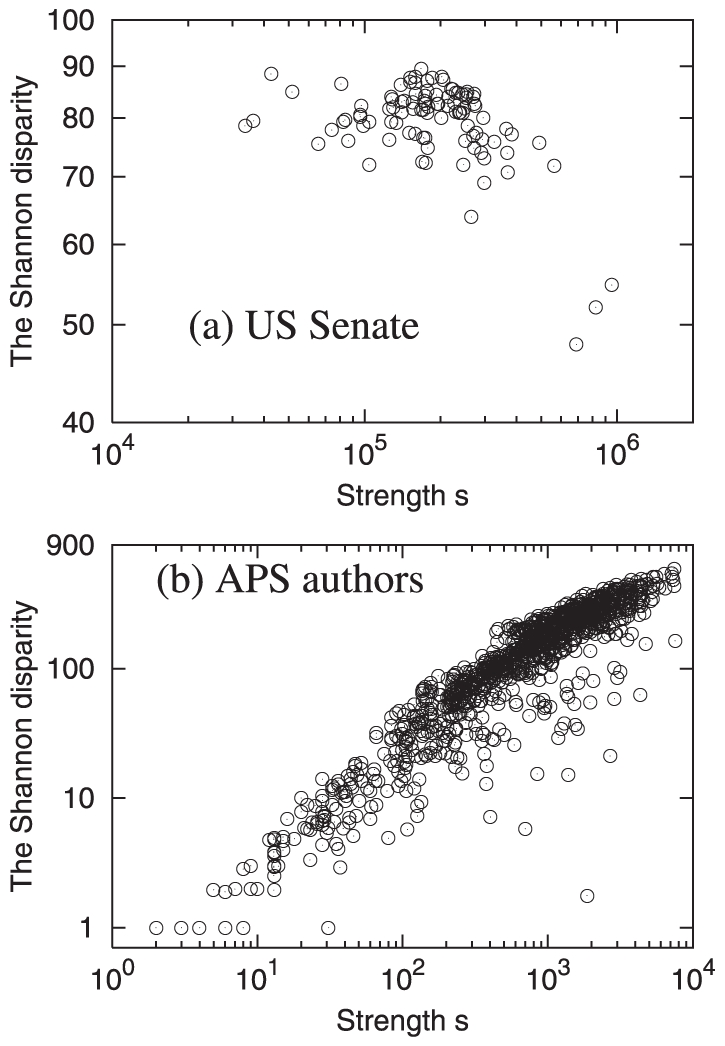
The scattered plots for the correlation between the strength 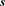 and the Shannon disparity 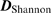 of each node. The correlations for (a) US Senate and (b) APS authors Google correlation network are shown. Graphs are drawn in the double logarithmic scale for easy visualization.

Figure 4 shows the correlation between the strength 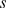 and the Shannon disparity 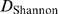 of each node for the two representative cases of the US Senate and APS authors. From the result, we can conclude that there are some senators with the very large strength and very heterogeneous Google correlation values with other senators, whereas the strength and the Shannon disparity is positively correlated for APS authors, which reflects the different attributes of political and academic communities. For instance, even the politicians meeting many other politicians may need to focus on the relationships with small groups of others sharing common interests with them, which might be related to the partisan politics [16], [17].

### Maximum Relatedness Subnetwork

As previously stated, the link density values of the Google correlation networks can be quite large compared with many sparse networks that have been previously investigated. Especially for the US Senate network, where almost every member is famous enough to appear on numerous webpages, almost all the possible pairs of senators are connected (a single webpage that is searched for each pair of senators can establish the link between any two senators). In such a case, beside the statistical properties, such as weight and strength distributions presented earlier, the mere figure of the weighted network itself can hardly give any visual clue for specific information about the structure of the community. In other words, there exist non-zero correlation values for almost all the pairs of nodes.

Econophysics has encountered similar situations in dealing with the financial time series correlations between companies or countries quite often and one way to circumvent the problem is the famous maximum (or minimum, depending on the definition of the correlation) spanning tree (MST) [18]. MST extracts the connected subtree (subnetwork without any loop) which maximizes (or minimizes) the sum of the weights on all the extracted links and one of the most popular methods of analyzing time series correlations in econophysics. Even for an unweighted network, one can extract MST of the network by assigning the edge betweenness centrality values as the links' weights so that the “skeleton” of the network is constructed [19].

In spite of the popularity of MST and its ability to select important interactions in many systems composed of pairwise correlations, there are a few drawbacks in the MST approach. First, the essential interactions do not need to connect all nodes as one giant component. In addition, MST uses the global rank of the weights as prime information for construction, and this might not be appropriate to access locally important interactions from the individual nodes' perspective.

We suggest a new approach, called the maximum relatedness subnetwork (MRS), as an alternative method to extract the essential interactions, instead of the conventional approach based on maximum spanning tree (MST) [18]. In MRS, for each node 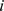, a directed link is connected from the node 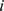 to the other node 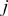 with which the node 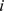 has its maximum correlation value. It is possible for a node to have more than one directed link in the case of the multiple nodes with the same maximum correlation value. In this way, for a network with an exactly uniform weight distribution, MRS is restored to the original network. MRS can resolve the problems of MST by not posing the restriction of “one connected component” and by using the locally maximum correlation values. Although it is difficult to assign intuitive meaning to MST, MRS has the clear interpretation of consecutively connecting to the maximally related nodes. For instance, a node's incoming degree in MRS shows how many of its neighbors consider the node as their most important partner and can be used as the measure of *reputation* or importance in the entire system. Furthermore, the directionality of MRS can yield new information about the asymmetry of the node pairs which is described below in detail.

The weighted social networks of our datasets constructed by the Google correlation values consist of undirected edges, as do most other social networks in the literature. This bidirectionality represents the mutual relationship in social networks and is easily understandable. The “mutual” relation, however, may not hold for the relationship given by the Google correlation. For example, the fact that a very famous person is connected with many members does not necessarily mean that she has many friends. Instead, it is possible that the members became connected to her just because she is famous and appears on many different webpages. Therefore, many asymmetric relationships (A is famous *mainly* because of B, but the opposite is not necessarily true) might appear, in the similar sense of the dependence relation between two authors in the collaboration network discussed in Ref. [20]. We believe that the directionality of MRS represents such asymmetric relationships or structures. For instance, if we consecutively “follow” the directed links in MRS, we can hierarchically reach links in the ascending order of weights. The link corresponding to the largest weight should be bidirectional by definition, although the converse is not always true. In addition, one can extend this concept further so that each node selects different number of nodes. One idea is that considering the Rényi disparity from the previous section as “effective” degree 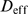 and choosing 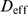 number of links with largest normalized weights.

## Results

### Maximum Relatedness Subnetwork of US Senate Network

**Figure 5:**
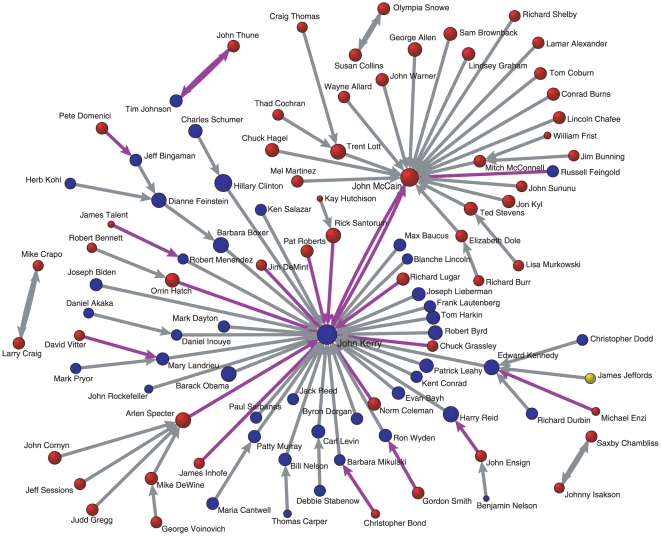
MRS of the US Senate Google correlation network, with the Google correlation values for May 4, 2006. The size of each node is proportional to the logarithm of the Google hit value [8]. The nodes' colors represent the political parties, i.e., blue for the Democratic party, red for the Republican party, and yellow for the independent Senator James Jeffords. The links are distinctly colored as positive (gray links) and negative (purple links) vote correlation in Eq. (6).

Figure 5 shows the MRS of US Senate in the 109th Congress. The most prominent senators are John Kerry and John McCain, who get many incoming links from other senators, implying that those numerous senators have the maximum Google correlation value with Senator Kerry or McCain. The division or community structure, reasonably consistent with the senators' political parties, is observed around the two prominent senators. Another property of MRS is that two adjacent senators are likely of the same state, e.g., Hillary Clinton and Charles Schumer from New York, George Voinovich and Mike DeWine from Ohio, *etc*. Other examples are Maria Cantwell and Patty Murray from Washington, Pete Domenici and Jeff Bingaman from New Mexico, Gordon Smith and Ron Wyden from Oregon, *etc*. All the four “isolated” mutually connected pairs are of this case: Johnny Isakson and Saxby Chambliss from Georgia, Mike Crapo and Larry Craig from Idaho, Susan Collins and Olympia Snowe from Maine, and Tim Johnson and John Thune from South Dakota. The last case, Tim Johnson and John Thune from South Dakota, is especially interesting because those two senators are mutually connected despite their difference in political parties. Therefore, MRS is not a random subset of a fully connected network but represents actual/relevant relationship between people. Some previous works about the community structures and interpretations for social networks among politicians are discussed in Ref. [16], [17]. We also successfully capture some aspects of this political network, and present from now on.

One can readily notice that almost all the senators around John McCain are the Republicans, whereas a relatively considerable number of non-Democratic senators are in John Kerry's side. The only Democratic Senator in John McCain's side is Russell Feingold, who has cosponsored the Bipartisan Campaign Reform Act, also known as McCain-Feingold Act, with John McCain. The likely connection between senators of the same state can explain such different compositions of communities. Among the 50 states, 21 states have two Republican senators, 15 states have two Democratic senators, and 13 states have one Republican and one Democratic senator. Therefore, a Democratic senator more likely serves with a Republican senator in a state than vice-versa, which can cause this kind of community structure. We consider the main factors setting the structure of MRS as the combination of the “global” effect based on the political parties and senators' individual fame, and the “local” effect based on the home states.

In this paper so far, we have focused on a snapshot of the Google correlation network. However, we can easily monitor the temporal changes by constructing the network on a regular basis, which is actually one of the most important advantages of our network construction scheme. In the following section, we use the US Senate network once again as an example of observing structural changes over time near an enormous political event, the United States Senate elections of 2006.

### Temporal Change of the US Senate Network near Election 2006

**Figure 6:**
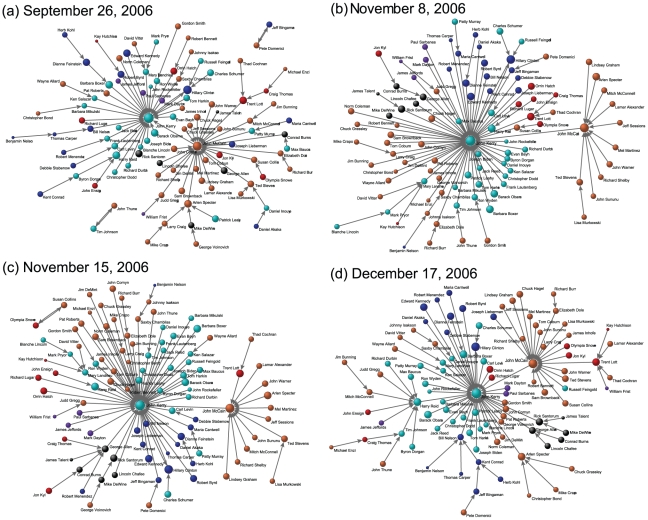
Four snapshots of MRS of the US Senate Google correlation network, near United States Senate elections 2006. The size of each node is proportional to the logarithm of the Google hit value. Senators are classified as re-elected Democrats (dark blue), Democrats not participating in the election (light blue), re-elected Republicans (dark red), Republicans not participating in the election (light red), Senators who failed to be re-elected (black; all Republicans), and Senators who retired (purple).

The United States Senate elections were held on November 7, 2006. We expected significant structural changes during this enormous political event, so we took four snapshots (September 26, November 8, November 15, and December 17) of the US Senate Google correlation networks near the elections, collecting the Google correlation values on the four specific days. Again, we observed the MRS of the network to infer the structural modification since the overall statistical properties such as weight and strength distributions, are similar for the four data. In Fig. 6, we present four snapshots of the MRS of the US Senate Google correlation network during the election period. A radical structural rebuilding of the MRS was observed during this period and was actually quite surprising because the webpages searched using Google are not always about the current news topics, but more like archives of the WWW from the entire historical database. The radical movements of senators in the MRS show that the dynamical webpages such as news articles, blog entries, and Wiki pages take a considerable amount of space on the WWW [21].

The most outstanding rearrangement in this period is a great movement of Senators from John McCain's side to John Kerry's side on November (Figs. 6(b) and (c)). The movement of the Republican election candidates (whether the candidate was re-elected or not) is particularly interesting. We suspect that one of the main reasons for this major change of the MRS is Senator John Kerry's “botched joke” about the Iraq War on October 30 and the following controversy [22]. Besides the MRS from Google correlation values we used, the impact of John Kerry's joke can also be checked in Google Trends, with which one can find how often people have searched certain topics on Google over time. If you type “John Kerry” on http://www.google.com/trends (last accessed on 6/2/2010), you will see a peak of search volume graph near November, 2006. We believe that many Republicans, who were at John McCain's side in the MRS before the elections (Fig. 6(a)) were involved in the controversy (with election candidates being most active), and their maximum Google correlation values moved from that with John McCain to that with John Kerry. After the elections, the impact of the controversy was relatively weakened and the MRS was reshaped again (Fig. 6(d)). Although we have only discussed the major movement tendency of senators and one possible cause, many other interpretations and further studies are possible. The techniques of Google correlation and MRS are widely applicable, and further progress will be achieved in the future.

### Aids to Obtain Further Specific Information

Relatedness, quantified by the Google correlation, could be the concept from either cooperation or competition. Google correlation values cannot solely distinguish whether a given relationship is friendly or hostile. External information can help us to specify the relationships in more detail, and, this this section, we show an example of such a specification with the US Senate network. The record of Roll Call Votes of the US Congress (http://thomas.loc.gov/home/rollcallvotes.html), which guarantees that every senator's vote is recorded, is used to elaborate relationships among senators.

With 642 Roll Call Votes of senators in the 109th Congress, we assign the *vote correlation* value 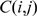 for every pair of senators 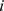 and 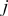 as follows:
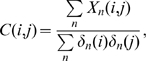
(6)where 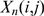 is 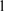 if senator 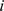 and 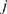 concurrently voted for or against the bill of the 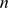
th Roll Call Vote and 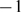 otherwise, and 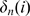 is 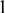 if Senator 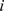 participated in the 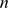
th Roll Call Votes and 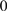 if Senator 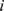 did not vote. We exclude the cases of unanimous votes to remove the effect of the entire Senate's opinion. Then, 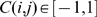 and measures the correlation of opinions of senator 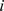 and 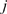.

Now we can infer the degree of cooperation with the vote correlation defined in Eq. (6). In Fig. 5, we distinguish the links among senators with the positive and negative vote correlation. From Fig. 5, we observe that the positive vote correlation is almost always given to the senator pairs from the same party and the negative vote correlation to the senator pairs from the different parties. Among all the senator pairs, only 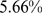 are from the different parties and have positive vote correlation value and 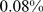 are with the same party and have negative vote correlation value, which implies the partisan polarization discussed in Ref. [23].

### Relationships between Two Groups: Bipartite Network Analysis

Investigating relationships via search engines is not restricted to a specific group of people. In addition, objects in a search query do not have to be restricted to people's names. We demonstrate this fact by investigating the relatedness between politicians and large corporations, revealing possible connections between politics and business. For sets of politicians, we selected 18 potential US presidential candidates in January 2008 and the 109th US Senators. The list of eighteen candidates are Hillary Clinton, Barack Obama, John Edwards, Dennis Kucinich, Joe Biden, Chris Dodd, Bill Richardson, Mike Gravel, Rudy Giuliani, Fred Thompson, John McCain, Mitt Romney, Mike Huckabee, Duncan Hunter, Tom Tancredo, Sam Brownback, John Cox, and Ron Paul. We chose the 100 largest corporations, as reported by *Fortune* [24] as the set of corporations.

The method of analysis is similar to the previous one, but in this case Google correlation values only *between politicians and corporations* are considered in a way to construct a so-called “bipartite network.” MRS is generated by collecting links from politicians to the corporations to which they are related most and vice-versa. Another measure introduced is the normalized Google correlation which represents the relatedness where the effect of fame is removed. This new measure is able to effectively prevent famous nodes from “dictating” the network. Normalized Google correlation between 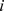 and 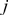 is defined as  = 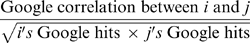. All the data for this analysis were collected in January 2008.

**Figure 7:**
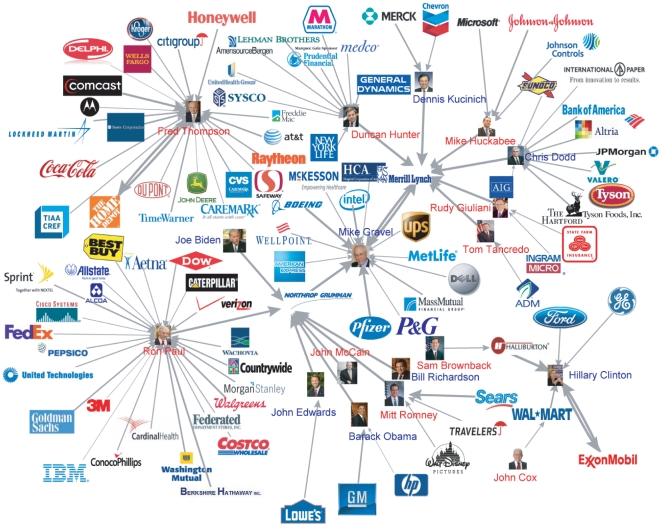
MRS from the bipartite network of the US presidential candidates and the 100 corporations. The Democratic candidates' names are colored as blue, the Republican candidates' names as Red, and the corporations as their logos. Normalized Google correlation values are used.

Figure 7 shows the MRS from the normalized Google correlation network of the US presidential candidates and the 100 corporations. John McCain, who has become the actual Republican presidential candidate at the time of writing, does not have many connections with large corporations in MRS. However, the only connected corporation with him is Northrop Grumman, which recently won the joint tanker contract to assemble the KC-45 refueling tankers for the US Air Force with EADS [25]. Because Senator John McCain once uncovered a corrupt effort by Boeing, which is Northrop Grumman's rival company [26], the connection looks interesting. The thick bidirectional connection between Senator Hillary Clinton and Exxon Mobil is likely from the large amount of money contributed to Senator Clinton from the corporation [27]. In similar ways, such analysis might give some hints for further investigation for the relationship between politics and business.

**Figure 8:**
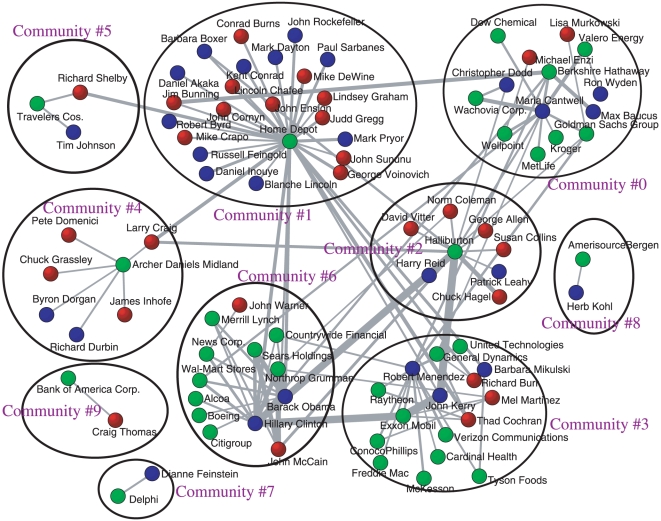
Community structure of the subnetwork. We keep only normalized Google correlation values larger than 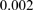, from the normalized Google correlation network of the 109th US Senators and the 100 corporations. The Democratic Senators are colored as blue, the Republican Senators as Red, and the corporations as green.

We also tried to elucidate community structures from the bipartite network between politicians and corporations as shown in Fig. 8. First we extracted the normalized Google correlation values between US Senators and the 100 corporations. Then we kept the link, whose Google correlation value is larger than 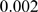, to obtain a sparser subnetwork for visualization. The community structure from the subnetwork was obtained by Newman's eigenvalue spectral method [28] and the modularity 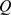 is 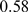, which might reveal the subunit of politics-business connections.

### Comparison with Real Social Networks

In this section, we provide evidence for the validity of social network construction by Google correlation values. We obtained a scientific collaboration network among the authors of papers citing the five key papers [29]–[33] in the network theory. The collaboration data was downloaded from ISI Web of Science, http://isiknowledge.com/ (last accessed on 6/2/2010). The 776 authors who wrote at least three papers were selected due to computational tractability. In this collaboration network, the pairs of authors who wrote the papers together were connected and the weights were assigned as the numbers of collaborated papers. To test the reliability of the Google correlation network among these authors, we constructed a weighted social network with the Google correlation values. To avoid the ambiguity of authors' name, the word “network” is added to the search query in this case, assuming most authors are related to the network research.

**Figure 9:**
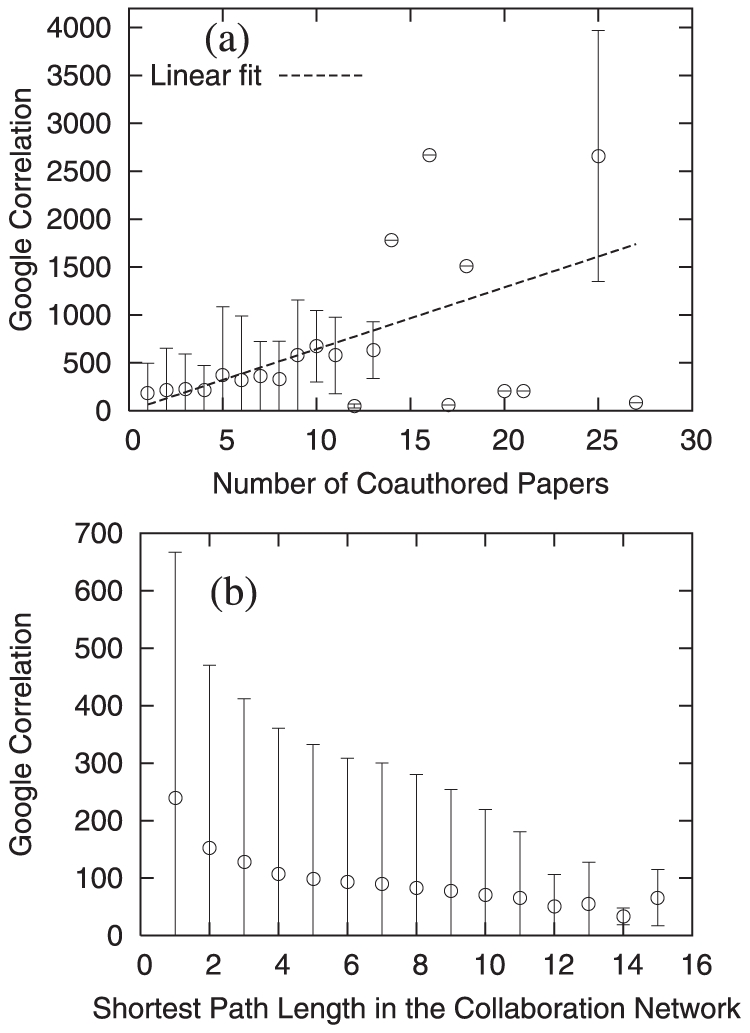
Comparison of a real social network and the Google correlation network. (a) The average Google correlation values for each number of collaborated papers. The error bars represent the standard deviations. The Pearson correlation coefficient between the number of collaborated papers and Google correlation values is 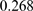 and the dashed line is the linear fit whose slope is 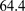. (b) The average Google correlation values for each value of the shortest path length among pairs of nodes in the collaboration network. The error bars represent the standard deviations. The Pearson correlation coefficient between the shortest path length and the Google correlation values is 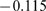.

The direct comparison between these two weighted networks (the collaboration network and the Google correlation network) is nontrivial, partly because of the enormous difference in the link density, i.e., the collaboration network is much sparser. Therefore, we suggest two schemes for comparison. First, we check the correlation between the weight in the collaboration network (the number of collaborated papers) and the Google correlation values for pairs of connected authors in the collaboration network. If the Google correlation network represents the true relatedness, we expect a positive correlation between the two quantities and Fig. 9(a) indeed shows a positive correlation. Second, regardless of whether two nodes in the collaboration network are directly connected or not, the Google correlation value and the shortest path length in the collaboration network for those two nodes are expected to be negatively correlated. Figure 9(b) confirms this expectation. Because the Google correlation value represents the relatedness of two authors, the larger the Google correlation value of the two authors, the nearer they are located in the collaboration network.

These correlations, of course, are not perfect. However, we suggest that the difference does not indicate the error or limitation of the Google correlation but reveals the actual difference between the collaboration and relatedness. Two authors can have large Google correlation value, even if they have never written papers together, if they work in the similar fields, show up at the same conferences many times, and thereby appear in the same “participant list” webpages of many conferences, for example. In summary, we have verified that our method actually reflects the structure of the real coauthorship network and have demonstrated the potential of our method.

Finally, we should mention caveats of our method. Many webpages are not under the quality control and may contain misleading or alleged facts. Therefore, our method should be considered as a *proxy* reflecting the real correlations. In other words, one has to be careful when dealing with the Google correlation data and note that any conclusions drawn from the analysis should be followed by accurate follow-up investigations, like genome-wide computational predictions followed by high-quality, small-scale experiments in biology.

However, in any case, we would like to emphasize that the Google correlation values can be the first, useful and exploratory step towards further investigations. We also want to point out that it is possible to flexibly customize the definition of the correlation measure for different purposes, for instance, by dividing the raw cooccurrence value by their Google hit values to get rid of their popularity effects whenever it is necessary, as suggested in the previous sections. Another way to customize our method is to use more specific search engines. For instance, for the coauthorship relations, one can count cooccurrences from Google Scholar, which indexes only the scholarly literature. Public relationships among politicians can be extracted more accurately by focusing on only the news articles. As an example, we constructed a network of Korean politicians by counting the number of news articles from a Korean online news service Naver(http://www.naver.com/), and demonstrated that the two clear groups in MRS well correspond to political parties and each party's leader/influential person possessing central position with many incoming links. In South Korea, the search engine Naver is more popular to the general public than Google, due to the many localized information and interface. Moreover, it deals with the Korean characters more appropriately than Google. So we use it for the analysis on the Korean politicians.

## Discussion

There is a tremendous amount of data on the Web, which can prove very useful if we harness it cleverly. Search engines are a basic device to classify such information and we have constructed social networks based on the Google correlation values quantifying the relatedness of people. We have systematically analyzed the basic statistical properties from the viewpoint of weighted network theory, introduced a new quantity called the Rényi disparity to represent the different aspect of the weight distribution for individual nodes, and suggested MRS to elucidate the essential relatedness. We have used the US Senate as a concrete example of our analysis and presented the results.

The concepts of the Rényi disparity and MRS introduced in this paper are not restricted to the Google correlation network, of course. The process of finding out “hidden asymmetry” of weighted links is applicable to other many weighted networks from various disciplines as well. In other words, such concepts can be interpreted as useful characteristics in different contexts. We have also compared a real scientific collaboration network with the social network constructed by our method introduced in this paper and discussed the result. The larger Google correlation values two authors have, the more papers they tend to have written together, causing them to appear to be “closer” in the scientific collaboration network.

Extracting information on the Web to construct networks makes it possible not only to obtain large networks with many participants, but also to monitor the change of such networks by collecting data on a regular basis. We have verified that the network structures do not change abruptly, partly because the Web plays the role of a digital “archive,” not a “newspaper.” However, during important events such as the elections for the United States Senate held in November 2006, the US Senate network was significantly reformed as we have discussed in this paper. If the webpages were classified into several categories such as news articles, blog articles, *etc.*, more information would be available. We hope that so-called Web 2.0 [7], [21], [34], [35] will significantly increase the possibility to obtain such classified information with ease in the future. The proper use of the Web and search engine in scientific research has already begun, for instance, in the research on the human tissue-specific metabolism [36], and we welcome other researchers who will join this movement in the future.
